# Endotoxin receptor CD14 in PiZ α-1-antitrypsin deficiency individuals

**DOI:** 10.1186/1465-9921-9-34

**Published:** 2008-04-21

**Authors:** Caroline S Sandström, Natalia Novoradovskaya, Corrado M Cilio, Eeva Piitulainen, Tomas Sveger, Sabina Janciauskiene

**Affiliations:** 1 Department of Clinical Sciences, Chronic and Degenerative Disease Research Unit, University Hospital Malmoe, Lund University, S-20502, Malmo, Sweden; 2 Stratagene, Agilent Technologies Company, 11011 North Torrey Pines Road, La Jolla, CA, 92037, USA; 3 Department of Clinical Sciences, Cellular Autoimmunity Unit, University Hospital Malmoe, Lund University, S-20502, Malmo, Sweden; 4 Department of Respiratory Medicine and Allergology, University Hospital Malmoe, Lund University, S-20502, Malmoe, Sweden; 5 Department of Clinical Sciences, Preventive Paediatrics, University Hospital Malmoe, Lund University, S-20502, Malmoe, Sweden

## Abstract

**Background:**

CD14, a receptor for lipopolysaccharides (LPS), is found in both a membrane-bound form (mCD14) and a soluble form (sCD14). It is suggested that sCD14 is mainly released from blood monocytes by serine protease-mediated shedding. Because α_1_-antitrypsin (AAT), an inhibitor of serine proteases, has been shown to regulate CD14 expression in human monocytes *in vitro*, we sought to investigate plasma levels of sCD14 and monocyte expression of mCD14 in subjects at age 30 years with normal MM and deficient PiZZ and PiSZ genotypes of AAT.

**Methods:**

Plasma levels of AAT and sCD14 were measured in 75 PiZZ and 34 PiSZ individuals with normal lung function identified from the Swedish neonatal AAT deficiency screening, and in 95 age matched PiMM controls. The mCD14 expression in monocytes from 9 PiZZ, 6 PiSZ and 11 PiMM subjects was analysed by FACS and Quantitative Real Time Reverse Transcription PCA.

**Results:**

As expected, plasma AAT concentrations were PiMM>PiSZ>PiZZ (p < 0.001). Plasma sCD14 levels were higher in PiZZ than in PiMM subjects (p < 0.01). The expression level of mCD14 was higher (1.89-fold) in monocytes isolated from PiZZ subjects compared to PiMM controls (p = 0.00189).

**Conclusion:**

This study is the first to show higher levels of plasma sCD14 and monocyte mCD14 expression in young, clinically healthy PiZZ AAT subjects.

## Background

CD14 is a multifunctional receptor constitutively expressed on the surface of various cells, including monocytes and macrophages, but it also occurs as a soluble form (sCD14) [1,2]. In normal serum, sCD14 is present in relatively high concentrations (>1 μg/ml). However, in different inflammatory and infectious diseases like sepsis and autoimmune diseases, characterised by the activation of monocytes and macrophages, highly elevated levels of sCD14 can be found [3,4]. *In vitro *and *in vivo *studies show that endotoxin (LPS) can induce sCD14 secretion and expression in hepatocytes [5,6]. In addition, after stimulation with various agents, an increased release of sCD14 is observed from monocytes. This suggests that sCD14 could be used as a marker of monocyte/macrophage activation [7,8]. The exact role of CD14 in normal and pathological situations is not completely known. Recent findings provide evidence that sCD14 expresses systemic anti-inflammatory effects and functions as a modulator of cellular and humoral immune responses [9-12].

Several studies have demonstrated a direct proteolytic effect of serine proteases, particularly neutrophil elastase (NE), on mCD14 resulting in the release of CD14 into the extracellular medium [7,13]. α_1_-antitrypsin (AAT), a major inhibitor of NE in the lower respiratory system, is thought to play an essential role in limiting host tissue injury at sites of inflammation [14]. The clinical importance of AAT is highlighted by an increased risk of developing chronic inflammatory disease, in particular chronic obstructive pulmonary disease (COPD), in individuals with severe AAT deficiency, PiZZ [15-18]. Severely decreased levels of AAT in subjects with AAT deficiency may result in increased activity of NE [19] with consequent proteolysis of immune cell membrane receptors, such as CD14, and release of soluble forms of those receptors. In addition, we have recently found that AAT also directly regulates CD14 expression and release in human monocytes *in vitro*. When cells were exposed for 18 h to a constant amount of AAT alone or in combination with LPS, CD14 and TLR4 mRNA expression was dramatically reduced compared to non-treated controls [20]. We hypothesize that AAT may play an important role in modulating sCD14 levels, both as an inhibitor of NE and as a direct regulator of CD14 expression and release, and, therefore, subjects with AAT deficiency may have higher CD14 expression than those with normal AAT levels. These considerations led us to examine plasma levels of sCD14 and blood monocyte mCD14 expression in clinically healthy young adults with PiSZ and PiZZ AAT deficiency relative to age and gender matched controls with normal PiMM AAT.

## Methods

### Study participants

This study included 204 individuals (98 males and 106 females) aged 30 (26–34) years. AAT-deficient subjects (n = 109; 75 PiZZ and 34 PiSZ) were recruited from the Swedish neonatal screening study [21]. Healthy age and gender matched controls (n = 95 PiMM) were randomly selected from the Swedish Population Registry. Data from physical examination and spirometry show that at the age of 30, individuals in this cohort have normal lung function [22]. In the PiMM group, 15 were current smokers and 16 ex-smokers; in the PiZZ groups 4 were smokers and 9 ex-smokers, and in the PiSZ groups 4 were ex-smokers. Informed consent was obtained from all individuals and the study was approved by the ethics committee, Lund University, Sweden and conducted according to the Helsinki Declaration.

### Sample collection

Blood was collected in tubes containing sodium heparin or ethylenediaminetetraacetic acid (EDTA) (B-D Vaccutainer System, USA) and centrifuged at 2000×g at 4°C for 10 min. Plasma was immediately separated and frozen at -80°C and stored until assayed.

### Isolation of monocytes

Blood monocytes were obtained from 26 individuals at age 32–34 years: PiMM (n = 11, 8 males and 3 females), PiZZ (n = 9, 5 males and 4 females) and PiSZ (n = 6, 4 males and 2 females). Monocytes were isolated using Ficoll-Paque PLUS (Pharmacia, Sweden) as described previously [23]. The cell number was determined by using an AC900EO Auto Counter (Swelab Instruments, AB, Sweden). The monocyte quantities in the preparations were greater than 70%. Cells were centrifuged at 300 g for 5 min, re-suspended in 2 ml fetal calf serum (FCS) containing 10% Dimethyl sulfoxide (DMSO) (Merck, Germany) and 5% D-Glucose-6-phosphate (Sigma, Germany) and stored in a freezer at -135°C (Revco, (Kendro), Bergman Labora, Sweden) until further analysis.

### Culture of monocytes

Cells were thawed and plated in 24-well cell culture plates (Corning Incorporated, USA) at a density of 1 × 10^6^cells/ml in RPMI 1640 (Gibco BRL, Life Technologies, Scotland) supplemented with 2 mM *N*-acetyl-l-alanyl-l-glutamine, 100 U/ml penicillin, 100 μg/ml streptomycin, 1% non-essential amino acids, 2% sodium pyruvate and 20 mM Hepes (Fluka, Chemie AG, Switzerland) without serum at 37°C in 5% CO_2_. After 75 min, non-adherent cells were removed by washing with PBS supplemented with calcium and magnesium. Monocytes were then cultured in medium for 18 h at 37°C in 5% CO_2_.

### Fluorescence-activated cell sorting (FACS) for CD14 expression

Monocyte CD14 expression was analysed by FACS. Cells were thawed and washed with PBS containing 2% FCS (Gibco BRL, Life Technologies, Scotland) and 0.01% NaN_3 _(VWR International Ltd., UK). Approximately 700,000 cells were incubated with 1.5 μg/ml FITC-conjugated anti-human CD14 antibody (Miltenyi Biotec, Germany) for 20 min in the dark at 4°C, washed and re-suspended in 300 μl PBS containing 2% FCS and 0.01% NaN_3_. FACS analysis was performed on a FACS Calibur (Becton Dickinson, USA) and data were processed using CellQuest Software (Becton Dickinson, USA).

### Monocyte lysate preparation

Human blood monocytes isolated from 26 individuals were thawed, washed with PBS twice and then lysed in the SideStep lysis and stabilization buffer (Stratagene, Agilent Technologies Inc., La Jolla, CA, USA) to a final concentration of 10.000 cells/μl. The SideStep buffer inactivates cellular nucleases and other enzymes, and the nucleic acids released into the buffer are stabilized and suitable for QRT-PCR analysis for at least 20 months when stored at -80°C.

### Quantitative Real Time Reverse Transcription PCA Analysis

20 μl of each SideStep cell lysate was diluted 40-fold using SideStep buffer to a final concentration of 250 cells/μl. 1 μl of cell lysate was used in 25 μl QRT-PCR reaction with SideStep™ II QRT-PCR Master Mix Kit, 1-Step (Stratagene, Agilent Technologies Inc., La Jolla, CA, USA), TaqMan primers and probes (B2M, catalog # Hs99999907_M1 and CD14, catalog # Hs00169122_g; Assay on Demand, Applied Biosystems, Foster City, CA, USA) on the Mx3000P Real-time PCR System (Stratagene, Agilent Technologies Inc., La Jolla, CA, USA). Each 25 μl reaction mix comprises 12.5 μl of 2× Brilliant II QRT-PCR master mix, 1.25 μl of TaqMan primers and probe mix, 0.375 μl of ROX reference dye, 0.1 μl of RT/RNase block mix, 9.77 μl of nuclease-free PCR-grade H_2_O and 1 μl of cell lysate. All reactions were run in triplicate using the following cycling parameters: 50°C/30 min, then 95°C/10 min followed by 40 cycles of 95°C/15 sec; 60°C/60 sec. *"No Reverse Transcription (RT) control" *was included for each target using 1 μl of cell lysate in order to confirm that there is no amplification of genomic DNA. Relative mRNA quantification was performed using the MxPro software application "Comparative Quantification" on the Mx3000P normalized to β-macroglobulin gene (B2M).

### Assays of sCD14

Plasma levels of sCD14 (n = 204) and culture supernatant levels of sCD14 in monocytes (n = 26) were measured using a commercially available sandwich enzyme-linked immunosorbent assay (ELISA) kits (R&D Systems, UK) according to the manufacturer's instructions. The minimum detection level for sCD14 was 250 pg/ml and the inter- and intra-assay coefficient of variation percentage (%CV) were 6.3% and 5.5% respectively.

### Plasma analysis of C-reactive protein (CRP)

The concentration of plasma CRP was analysed (n = 199) at the Department of Clinical Chemistry in Malmoe (Malmoe University Hospital, Sweden) using high sensitivity method with minimum detection level of 0.2 mg/l.

### Analysis of plasma AAT

Plasma AAT was analysed by nephelometry at the Department of Clinical Chemistry (Malmoe University Hospital, Sweden) or by rocket-immunoelectrophoresis described by Laurell [24]. In brief, aliquots of plasma were run for 1.5 h at 200 V on 1 mm 0.9% w/v agarose gels containing 11 mg/l anti-human AAT antibody (DakoCytomation, Denmark). Gels were pressed between filter papers and dried before staining with Coomassie Blue. To quantify AAT, the distance between the tip of the rocket-shaped immuno-precipitates and the application well, was measured. Standard curves were generated by serial dilutions of a standard (Seronorm, Sero AS, Norway) that was run in parallel to samples on every gel. The coefficient of variation percentage (%CV) for the inter- and intra-batch variability was 7.9% and 5.8% respectively.

### Statistical analysis

The data were analysed using the Statistica software (Series1203b, version 6.1 for Windows, Statsoft^®^, USA), the SPSS software (version 12.0.1, for Windows, SPSS Inc., USA). All variables were analysed for the normal distribution by Kolmogorov-Smirnov test.

The one-way ANOVA combined with a multiple-comparisons procedure (Scheffe multiple range test) was used to compare differences in means for both sCD14 and mCD14 and the unpaired Student's *t*-test was used to investigate differences in plasma sCD14 between genders within the three groups (MM, ZZ and SZ). The Student's *t*-test with correction for multiple comparisons was performed to evaluate the differences in CD14 mRNA expression level in MM, SZ and ZZ monocytes. Plasma AAT levels were analyzed by the Kruskal-Wallis method for comparisons between more than two groups, and if significant, groups were compared by using the Mann-Whitney U test and corrected for multiple comparisons (Bonferroni). Tests with p < 0.05 were considered to be significant and data are presented as mean ± SEM or SD.

## Results

### Plasma levels of AAT, sCD14 and CRP

As expected, the levels of plasma AAT were significantly lower in ZZ subjects compared to both SZ and MM (p < 0.001). In agreement with previously published data [25] the concentrations of AAT were higher in MM and ZZ females than in males (p < 0.05 and p < 0.01, respectively). Interestingly, plasma sCD14 levels were higher in ZZ than in SZ (p < 0.05) and MM (p < 0.01) groups, but there was no difference in sCD14 levels found between MM and SZ groups. There was no difference in plasma sCD14 levels between males and females within the groups (Table 1). Increased levels of CRP indicate different inflammatory conditions. No significant difference was observed in mean CRP values between MM, ZZ and SZ (data not shown). Mean CRP was below the reference value (<3 mg/l) in all three groups.

**Table 1:** Plasma concentrations of sCD14 and AAT.

Pi group	sCD14 (μg/ml)			AAT (mg/ml)		
	Total	Males	Females	Total	Males	Females
MM (n = 95)	1.26 ± 0.03	1.25 ± 0.05 (n = 38)	1.26 ± 0.03 (n = 57)	1.45 ± 0.04	1.35 ± 0.04^II^ (n = 38)	1.51 ± 0.06^II^ (n = 57)
ZZ (n = 75)	1.40 ± 0.04^*, ‡^	1.38 ± 0.05 (n = 43)	1.43 ± 0.06 (n = 32)	0.24 ± 0.01^†,§^	0.22 ± 0.01** (n = 43)	0.26 ± 0.01** (n = 32)
SZ (n = 34)	1.24 ± 0.04	1.24 ± 0.05 (n = 17)	1.24 ± 0.06 (n = 17)	0.61 ± 0.03^†^	0.57 ± 0.02 (n = 17)	0.66 ± 0.05 (n = 17)

Abbreviations used: n, number of cases; sCD14, soluble CD14; AAT, α_1_-antitrypsin.

Results are mean ± SEM.

* p < 0.01 vs MM,

^† ^p < 0.001 vs MM,

^‡ ^p < 0.05 vs SZ,

^§ ^p < 0.001 vs SZ,

^II ^p < 0.05 between gender within group,

** p < 0.01 between gender within group.

### Monocyte mCD14

**Figure 1:**
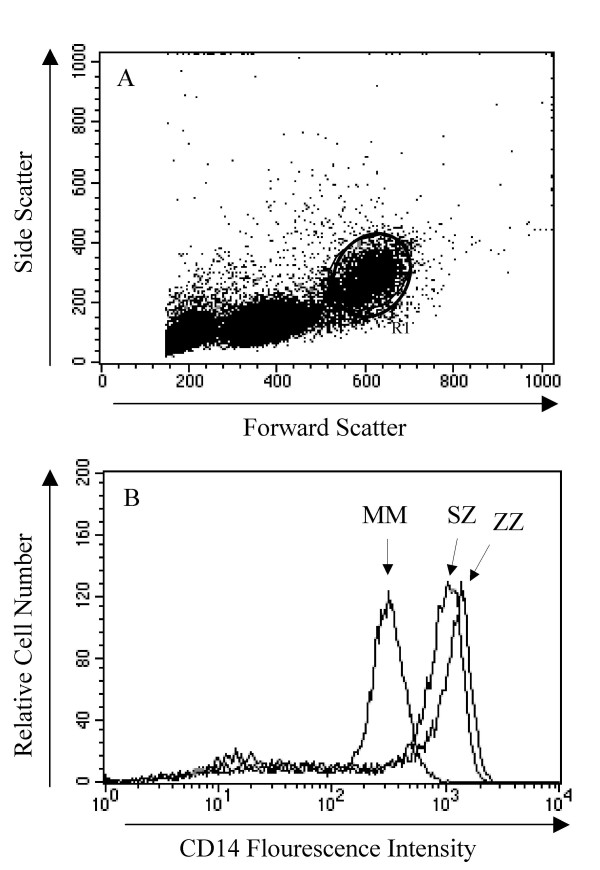
**A and B**. (A) representative selection (R1) of CD14+ cells from the total leukocyte population; and (B) total CD14 fluorescence intensity in the selected CD14+ population. Cells were stained with a CD14 FITC-conjugated antibody and analysed with FACS (as described in methods). Monocytes obtained from AAT deficient individuals (ZZ and SZ) showed a tendency to express more membrane bound CD14 than MM cells. Data show one representative analysis from each group.

Since CD14 is predominantly expressed on the surface of monocytes, we investigated the levels of mCD14 on blood monocytes isolated from PiMM (n = 11), PiZZ (n = 9) and PiSZ (n = 6) individuals. We first evaluated the distribution of all measured cell populations that comprise the peripheral blood mononuclear cells (PBMCs). As shown in Figure 1A, lymphocytes and monocytes were differentiated by characteristic side and forward light-scattering properties and confirmed by CD14 staining. However, we found no differences among the PiMM, PiZZ and the PiSZ individuals when comparing the fraction of CD14+ cells with the total number of PBMC (Fig. 1A). We found no statistical difference in the expression of monocyte mCD14 among the groups, but the mean value of the fluorescence intensity (MFI) was 878.1 ± 51.2 and 742.7 ± 47.1 for PiZZ and PiSZ vs 714.5 ± 65.7 for the PiMM, suggesting a higher expression of mCD14 on monocytes from deficiency subjects (Fig. 1B).

### QRT-PCR analysis of mCD14 expression in monocytes

**Figure 2:**
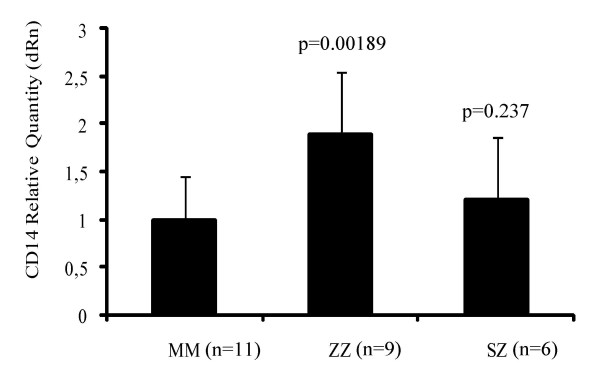
The relative quantity of CD14 mRNA expression in normal (MM) and deficient (ZZ and SZ) monocytes after gene normalization using the "Comparative Quantification" application of MxPro software. CD14 expression level was significantly higher in the ZZ group compared to normal. Each bar represents mean ± SD of triplicate QRT-PCR reactions.

The expression level of CD14 mRNA was also evaluated by QRT-PCR in blood monocytes isolated from PiMM (n = 11), PiZZ (n = 9) and PiSZ (n = 6) individuals. To determine the amplification efficiencies of both reactions, a standard curve was generated using two-fold serial dilutions of the SideStep cell lysate. The amplification efficiencies of 100.8% and 100.6% were obtained for CD14 and reference B2M targets, respectively. No amplification signal was observed in "*no RT control*" reactions for both gene targets that confirmed that there was no amplification of genomic DNA. The threshold cycle differences (ΔCt) between the gene of interest (CD14) and the normalizer (B2M) were calculated for each sample and an average of ΔCt for each group of individuals was used to evaluate fold differences in CD14 expression using the delta-delta Ct (ΔΔCt) method. The relative quantity chart of CD14 mRNA expression in normal PiMM (n = 11) and deficient PiZZ (n = 9) monocytes showed that the CD14 expression level was significantly higher (1.89-fold) in PiZZ monocytes relative to normal PiMM (p = 0.00189). No significant difference was found between PiSZ and PiMM monocytes (p = 0.237) (Fig. 2).

### Levels of sCD14 in monocyte culture supernatants

**Figure 3:**
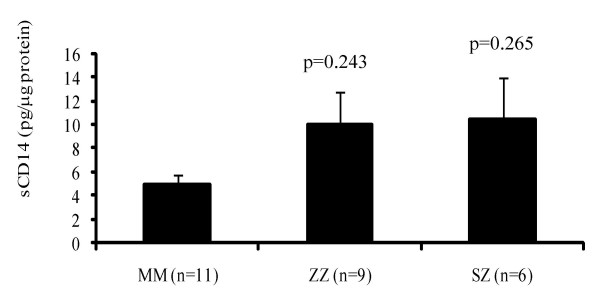
The secretion of sCD14 from monocytes incubated for 18 h at 37°C. Monocytes isolated from ZZ and SZ subjects demonstrated a tendency to release higher levels of sCD14 than MM cells. Each bar represents mean ± SEM.

Soluble CD14 levels in overnight monocyte-conditioned medium for PiZZ (n = 9), PiSZ (n = 6) and PiMM (n = 11) subjects were measured. As illustrated in Figure 3, the amount of sCD14 released by monocytes from PiZZ and PiSZ subjects (mean value 10.1 ± 2.7 and 10.5 ± 3.5 pg/μg protein, respectively) was higher than from PiMM monocytes (5.0 ± 0.9 pg/μg protein), but did not reach statistical significance (p = 0.243 and p = 0.265, respectively).

## Discussion

The Z mutation of AAT leads to a conformational misfolding of the protein with accumulation in the endoplasmatic reticulum of hepatocytes and reduced export from the liver [26]. The consequent lack of circulating AAT in individuals with severe PiZZ AAT deficiency may lead to development of premature pulmonary emphysema and COPD. Pulmonary symptoms occur in the 3rd to 5th decade of life [27] and the progression of lung disease is markedly accelerated by cigarette smoking [28]. Because of both the lack of knowledge about the inflammatory processes in COPD and a lack of established biomarkers, it is not possible today to detect sub-clinical lung obstruction in time for preventive physician intervention that could limit lung tissue damage. Therefore, many of the COPD cases with AAT deficiency are undiagnosed or diagnosed too late. From 1972 to 1974, 200,000 Swedish newborns were screened for AAT deficiency. Those with severe (PiZZ) and moderate (PiSZ) deficiency having plasma levels of AAT that are 10–15% and 30–40% of the normal levels have been followed prospectively [21]. The important aims of this project were to study the natural history and pathophysiology of AAT deficiency in childhood and adolescence, and to prevent lung disease. Follow-up results of PiZZ and PiSZ individuals at age 30 indicate retention of normal lung function but with a higher prevalence of respiratory symptoms compared to age matched PiMM controls [22]. Currently there are no reliable clinical and/or biochemical parameters that allow us to predict who of those individuals will get COPD and emphysema. Therefore, studies aiming to improve our understanding of the basic mechanisms behind the development of COPD and to translate this to the diagnosis and/or the treatment are of great importance. We believe that the examination of the possible difference in cellular protein expression and the release profile between clinically healthy Z and M AAT subjects may improve our understanding of the mechanisms involved in the development of COPD.

To our knowledge, this study is the first to demonstrate significantly higher sCD14 levels in plasma from 30-year old PiZZ deficiency subjects compared to age matched PiSZ individuals and PiMM controls. It is important to note that this finding was not related to the levels of the inflammatory marker CRP nor to the smoking habits of our study participants. Our results also indicate that ZZ and SZ monocytes have higher surface expression of mCD14 and that these monocytes cultured over night release larger amounts of sCD14 than MM monocytes. Whether higher plasma levels of sCD14 and higher mCD14 expression in blood monocytes in the AAT deficient individuals reflect specific pathophysiological changes in these individuals and may be used as specific marker of inflammation remains to be proven.

Several studies have demonstrated a direct proteolytic effect of NE on mCD14 [13]. Individuals with severe AAT deficiency arising from lower plasma levels and decreased activity suffer a shortfall of NE inhibitor capacity. According to Ogushi and colleagues, Z type AAT-elastase complexes are less stable than M type AAT-elastase complexes and, compared to active M type AAT, active Z type AAT requires twice as long to inhibit neutrophil elastase [29]. Therefore, Z AAT individuals have uncontrolled elastase activity and increased susceptibility to emphysema as a result of these lower levels and dysfunction of the AAT protein. This supports the concept that lack of AAT may be the reason for the higher sCD14 shedding in subjects with severe AAT deficiency. Different assays usually fail to discriminate between free active elastase and elastase-inhibitor complexes. These limitations have hampered our investigations of the direct relationship between plasma levels of free NE and sCD14 in AAT deficiency subjects.

It is not yet known whether monocytes and macrophages are the major sources of sCD14, since hepatocytes also have been shown to produce sCD14 [5,6]. Circulating AAT is primarily synthesized in the liver and secreted into the bloodstream [30]. Hepatocytes from Z AAT individuals may express greater amounts of cell-associated CD14 protein, and therefore more of the sCD14 is released resulting in an inverse link between plasma levels of Z AAT and sCD14 in AAT deficiency. Further studies will be needed to test these speculations.

The biological function of sCD14 is so far not clear. *In vitro*, an excess of sCD14 is shown to inhibit LPS binding to mCD14 and hence block cellular activation [11,31]. It has also recently been shown that the response to low-dose LPS instilled intratracheally is completely dependent on CD14, whereas at higher doses the response is also dependent on CD11b [32]. Grunwald and collaborators have shown that binding of LPS to monocytes and LPS-induced cell activation are abrogated by an exogenously added high dose of sCD14 [33]. Moreover, injection of sCD14 has been shown to decrease endotoxin-induced mortality in a murine model [31]. It has also been demonstrated that intravenous injection of an anti-CD14 antibody attenuates LPS-induced lung injury [34]. It also appears that CD14 may be critical to LPS-induced airway disease and that macrophage CD14 is sufficient to initiate neutrophil recruitment into the airways but that CD14 may need to interact with other cell types [35].

## Conclusion

CD14 is a pattern-recognition receptor that plays a central role in innate immunity and directs the adaptive immune responses. We for the first time demonstrate that clinically healthy young adults with PiZZ AAT deficiency have higher plasma sCD14 levels, show increased sCD14 release and higher mCD14 expression in peripheral blood monocytes as compared to age matched PiMM subjects. Our findings, therefore, argue strongly for further studies on relationship between CD14 expression, AAT deficiency and increased susceptibility of PiZZ subjects to develop emphysema.

## List of abbreviations

AAT: α_1_-antitrypsin; COPD: Chronic obstructive pulmonary disease; CRP: c-reactive protein; LPS: Lipopolysacharride; sCD14: Soluble CD14; mCD14: Membrane CD14; NE: Neutrophil elastase.

## Competing interests

The authors declares that they have no competing interests.

## Authors' contributions

All authors have contributed to the manuscript. SJ designed the study. CS and NN carried out the experiments. CC was responsible for FACS experiments and data interpretation. CS, NN and SJ prepared figures and wrote the manuscript. TS and EP helped with the proof reading of the manuscript. All authors have read and approved the final manuscript.

## Funding

This work was supported by grants from Zoega Foundation, Swedish Research council, MAS Foundation, Lundstrom Foundation and Heart Lung Foundation.
